# Synthetic microbial communities^☆^

**DOI:** 10.1016/j.mib.2014.02.002

**Published:** 2014-04

**Authors:** Tobias Großkopf, Orkun S Soyer

**Affiliations:** School of Life Sciences, University of Warwick, United Kingdom

## Abstract

While natural microbial communities are composed of a mix of microbes with often unknown functions, the construction of synthetic microbial communities allows for the generation of defined systems with reduced complexity. Used in a top-down approach, synthetic communities serve as model systems to ask questions about the performance and stability of microbial communities. In a second, bottom-up approach, synthetic microbial communities are used to study which conditions are necessary to generate interaction patterns like symbiosis or competition, and how higher order community structure can emerge from these. Besides their obvious value as model systems to understand the structure, function and evolution of microbial communities as complex dynamical systems, synthetic communities can also open up new avenues for biotechnological applications.

## Highlights

• Microbial interactions and system function are two ways to study communities.
• Natural microbial communities are difficult to define and to study.
• Synthetic microbial communities are comprehensible systems of reduced complexity.
• Synthetic communities keep key features of natural ones and are amenable to modelling.
• Synthetic microbial communities are gaining importance in biotechnology.

**Current Opinion in Microbiology** 2014, **18**:72–77

This review comes from a themed issue on **Cell regulation**

Edited by **Cecília Maria Arraiano** and **Gregory M Cook**

For a complete overview see the Issue and the Editorial

Available online 14th March 2014

1369-5274/$ – see front matter, © 2014 The Authors. Published by Elsevier Ltd. All rights reserved.

**http://dx.doi.org/10.1016/j.mib.2014.02.002**

## The challenges of understanding natural communities

Recent years have seen a surge in the analysis of microbial communities thanks to the development and cost-reduction in sequencing technologies. The continuing application of these has led to the characterisation of species diversity in different natural communities from the oceans to the human gut [1]. Despite this rapid increase in available metagenomic datasets, however, there are still significant limitations in answering the fundamental ecological and evolutionary questions surrounding natural microbial communities [2–4]. In particular, we still lack a clear understanding of the molecular and ecological bases of community-level functions, and the potential trade-offs in the extent, stability and robustness of such functions and other community properties (e.g. diversity, structure, size).

While even the simplest of the natural microbial communities characterised to date contain tens to more than thousands of species [5], it is usually not possible to experimentally verify which species in such characterisations are actively part of the community, or are performing key functions. The assembly of natural microbial communities might also depend on dispersal of potential seed organisms, as well as on environmental selection and species sorting [6], the effects of which would be difficult to control or characterise. Further, the influence of each of such drivers on the composition of a community can vary according to the ecosystem and temporal scale of observation. For example, open systems like wastewater treatment plants are found to be dominated by near random colonisation effects [7,8], while more enclosed systems like the gut of higher animals seem to lead to a more stable community over time after an initial period of community assembly [9]. The known limitations in detecting rare species with sequencing approaches [5,10] create a further challenge in dissecting the relation between species composition and community function and dynamics.

## Synthetic microbial communities as model systems

A promising way to overcome the difficulties associated with studying natural communities is to create artificial communities that retain the key features of their natural counterparts. These can then act as a model system to assess the role of key ecological, structural and functional features of communities in a controlled way. Here, we define a synthetic community as one that is created artificially by co-culturing of select (two or many) species under a (at least initially) well-defined media. While the individual species in such a community can be further engineered, we do not require presence of synthetically engineered species for a community to be defined as synthetic. Towards an increased understanding of natural communities, these synthetic communities can be utilised in two general ways, which we classify as function-based and interaction-based.

### Function first: studying communities with determined function

This top-down approach focuses on determining a functional definition for a community first and then using this definition to characterise community structure and dynamics in detail. The value of having a defined ‘function’ for a microbial community allows us to measure the extent and stability of this function and, perhaps more importantly, gives us a way to compare different communities with the same function.

Within this approach, ‘natural’ microbial communities utilised in biotechnology are of interest, as they are operated under relatively well-defined conditions and where the production or degradation of a target substance can act as an objective measure of performance. In anaerobic digestion and wastewater-treatment plants, for example, production of biogas or reduction of toxic organic compounds can be taken as the community function. This allows long-term measuring of parameters such as functional stability and performance, and correlation of these with community composition [11]. While promising, these approaches rely on our ability to accurately measure species diversity in a complex community, which is known to be limited [5,10].

This limitation can be surpassed by directly manipulating the level of diversity in a carefully designed synthetic community, which allows exploring the relation between community structure and function in a controlled way. For example, by defining mercury removal as the community function and creating communities with different levels of diversity, Von Canstein *et al.* found an improvement in functional efficiency with increasing diversity [12]. Similarly, Kassen *et al.* used indirect control on radial diversification of *Pseudomonas fluorescens* to show that productivity (i.e. the rate of production of organic matter by a community) displays a unimodal relation to diversity, peaking at intermediate levels of diversity [13].

The functional approach to communities is not only useful to study the relations between key properties such as diversity, function, and stability, but also allows deriving a mechanistic understanding of community structure. In the context of anaerobic digestion, the definition of community function as conversion of complex organic matter into methane has allowed a mechanistic understanding of the underlying communities as species assemblies interlinked through the different levels of the degradation process [14]. This mechanistic understanding allows developing a sense for key species in the system, as well as defining potential bottlenecks and control points.

### Interactions first: key metabolic interaction patterns as determinants of community structure and dynamics

In this bottom-up approach the focus is on identifying common interaction patterns and processes among bacterial species, with the expectation that these could be key determinants of the overall community structure and dynamics. Species interactions in microbial communities can be either metabolism-based or be driven by social traits. To date, social interactions among microbes has attracted attention and several good reviews have summarised the study of these interactions in synthetic as well as natural microbial communities [15^•^,16–18]. Here, we emphasise the role of metabolism in driving the species interactions in microbial communities.

**Figure 1:**
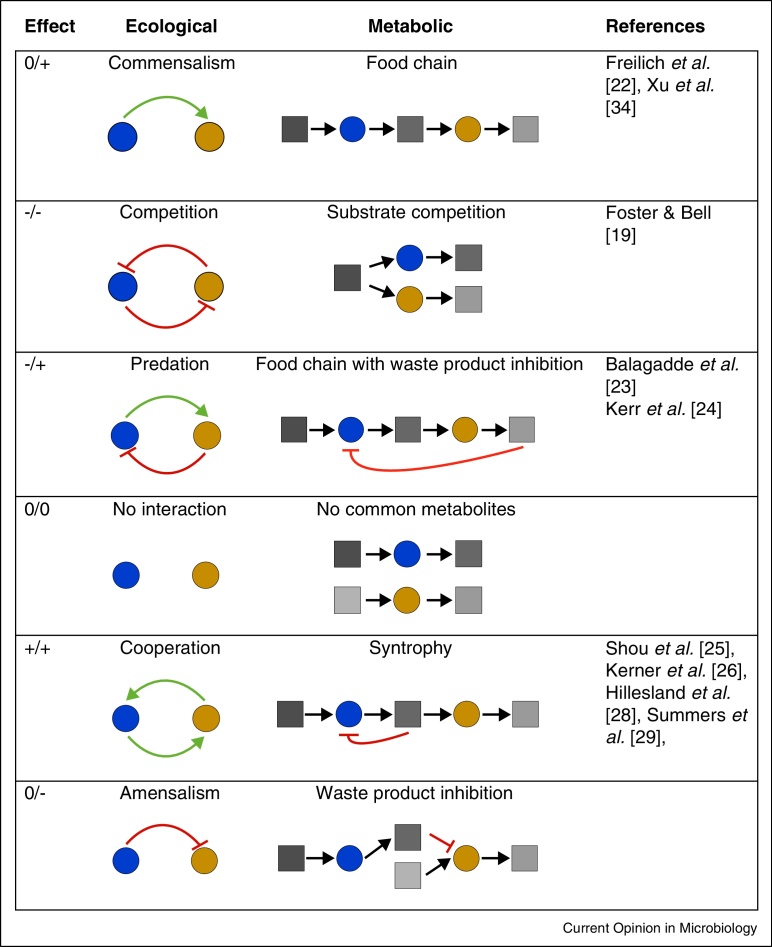
The six basic motifs of microbial interactions. Blue and yellow circles denote different microbial strains respectively, while boxes represent metabolites. Stimulating and inhibitory interactions mediated by species traits or metabolites are indicated with red and green arrows respectively. References are to studies in which synthetic microbial communities corresponding to the given interaction type are developed. In case of a syntrophy, the first microbe in the food chain (blue circle) is inhibited by the accumulation of its own waste product in the environment (mainly via thermodynamic limitations). This inhibition is relieved by the second microbe (yellow organism), which uses the waste product of the first microbe as a food source. Hence, both organisms benefit from the presence of the other.

The commonly considered social interactions of competition and cooperation are only two of all possible interactions among microbial species. The net effect of an organism A on a second organism B can be neutral, positive or negative, resulting in the number of possible mutual interactions between two organisms to be 9. Since the directionality of a reaction is not of interest for the type of interaction (i.e. +/− = −/+), there are six basal interaction patterns that make up the minimal interaction motifs in microbial communities (Figure 1). While each one of these interactions can be potentially driven through social traits of the involved species [19] or environmental factors like patchiness (i.e. local differences in species abundance) [20^••^,21], we argue that the simplest driver is metabolic interactions. In Figure 1, we provide the mapping of all possible interaction patterns between two species into an ecological and a corresponding metabolic representation (i.e. the communication between species via their central metabolic products). A food chain for example can be seen as a commensal interaction, where organism B lives off of the waste of organism A, who in turn is not affected by the interaction (0/+). Similarly, competition arises naturally when two organisms A and B utilise the same substrate, and an intense form of cooperation emerges, when each of the partners gain by the metabolic reactions of the other (i.e. syntrophy). While the combination of two different strains allow for six possible interaction states, a system with three strains has already 729, one with four strains 531441 possible states of microbial interactions. Since such combinatorial explosion of the number of possible interaction states quickly reaches large numbers with only a few species, the challenge is to find key motifs that are over-represented in nature or that can have significant percolating effects at the community level (e.g. stabilizing or de-stabilising interaction motifs, motifs driving oscillatory or chaotic dynamics).

It is hypothesised for example that cooperative interactions among the members of the community could be a major driver of community function and structure [15^•^]. This idea motivates recent computational and experimental studies on determining pairwise interaction patterns among different microbes. These studies, while preliminary, indicate that cooperation might not be the dominant interaction pattern among microbial species. A metabolic modelling study of pairwise combinations of 118 isolated bacterial strains, for example, showed that positive interactions are unidirectional in most cases, hence falling in the commensal category [22^•^]. Likewise, an experimental study by Foster *et al*. found the interactions between two randomly selected members of a beech tree hole community to be mostly competitive [19].

The potential role of the presence or absence of cooperation on community dynamics can also be analysed by synthetically engineering cooperation. Cooperation between two microbial strains can either be engineered by knocking out different essential genes in each of the strains [23,24], by specific manipulation of the environment [25^•^,26] or by selecting partner organisms that are bound to cooperate by thermodynamics, for example, when one organism produces a waste product that inhibits its own metabolic reaction when accumulating in the environment [27,28]. Besides cooperation, several other interaction motifs have also been tested with synthetic communities featuring synthetically engineered species. For example, Balagaddé *et al.* used engineered strains of *Escherichia coli* to analyze species dynamics resulting from predator-prey interactions (corresponding to a food chain with waste inhibition in metabolic-terms) [29], while Kerr *et al.* used a similar approach to create a three-species interaction pattern corresponding to a rock-paper-scissors game [30].

While such applications of synthetic biology in constructing synthetic communities were so far driven by an ecological view of interaction motifs, we believe that the construction of interactions using a metabolism-based view could be highly useful for several reasons. Firstly, ‘metabolic interaction motifs’ could be more easily linked to natural systems. Naturally prevalent motifs could be potentially identified by combining metagenomic and species co-occurrence analyses, with the graph-theoretical analyses of metabolic network models of each species. For example, the concept of ‘seed sets’, the set of metabolites a microbial species needs to acquire from the environment [31], could be combined with the knowledge of species’ co-occurrence patterns [32] to predict potential metabolic interactions. Similarly, creating combined metabolic models of different species connected through metabolite exchange [22^•^,26] can allow the identification of metabolic interaction motifs among such species. Secondly, metabolic interaction motifs can be identified, or expected, from the functional characterisation of the microbial community under study. For example, it is well known that methane-forming communities feature methanogens, which are usually found under syntrophic metabolic interactions (i.e. cooperation) with other species [33]. Finally, and perhaps most importantly, metabolic interaction motifs are easily converted into dynamical models, for example using ordinary differential equations, and allow direct comparison of model outcomes and measurements of metabolites and populations. This provides characterisation of the resulting species dynamics, as done successfully in the case of syntrophic and cyclical metabolic interactions [34,35]. Thus, the metabolism-based view allows for the creation of both theoretical models and experimental implementations of the simple interaction motifs found in microbial communities.

Synthetic microbial communities can also be used to combine the function-first and interaction-first approaches summarised above. This can be achieved by creating synthetic communities where both species composition and community function are defined. The resulting systems provide important insights into the relation between community function, structure and dynamics. Examples of this combined approach include those that focused on characterising species interactions and stable performance in synthetic communities for cellulose degradation [25^•^,36].

## Application of synthetic communities

We believe that understanding metabolic interactions in microbial communities and the ability to artificially stabilise interactions between microbes will eventually allow us to better understand and harvest the full metabolic potential of the microbial world.

Several challenges, however, need to be overcome for the rational engineering of microbial communities. The foremost of these is to achieve media compositions that can maintain multiple species. Given that media composition is a dynamic parameter of the system, linked to population dynamics, further development of metabolic as well as population dynamics models [34,35,37,38] need to be combined with experimental work. Such models can then be further refined through experimental data, but achieving this will require high-throughput data collection methods. Population dynamics measurements through rapid sequencing or reporter-based methods need to be established for synthetic communities. Long-term monitoring and culturing of communities will require further development of mini-bioreactor technologies, with microfluidics based approaches being most promising [39^••^,40].

We expect that as these challenges are overcome and rational engineering of synthetic communities becomes more mainstream and high-throughput, synthetic microbial communities will find more applications both in microbial ecology and in biotechnology. Especially with respect to biotechnology, the synthetic microbial community concept has a great potential for the future [41]. A rational design and assembly of microbial specialists to perform a given function allows for the differential regulation of subfunctions by controlling the density of the microbes that perform them [24]. This might be a more promising approach than synthetic manipulation of a single synthetic strain, especially when complex tasks are required [42–44], and consequently it is already finding its way into fields like biofuel production [45,46].

## Conclusion

Synthetic microbial communities are abstractions of natural systems that allow the in detail study and analysis of the fundamental building blocks and processes that compose a microbial community. Both the top-down and bottom-up approaches summarised above have yielded important results on simple communities and are paving the way for the assembly of higher order communities. With the aid of novel approaches such as micro bead encapsulation of a small number of microorganisms [39^••^,47] microfluidic chip technology [40] or the sequential layering of microbes onto a synthetic biofilm [48], we expect the construction of synthetic communities to get easier and potentially becoming high-throughput.

Research into synthetic microbial communities is witnessing a re-incarnation after the first attempts in creating co-culture and tri-culture about several decades ago [49,50]. We feel that this time they are here to stay as an important part of the toolkit of microbial ecology and biotechnology.

## References and recommended reading

Papers of particular interest, published within the period of review, have been highlighted as:

• of special interest
•• of outstanding interest
